# A Scoping Review on Sex and Gender Differences in the Adverse Health Outcomes of Individuals with Sickle Cell Trait

**DOI:** 10.1089/whr.2024.0092

**Published:** 2024-12-05

**Authors:** Angela K. Phillips, Laura Segovia, Alicia A. Livinski, Candy Wilson, Arun S. Shet, Margaret Bevans

**Affiliations:** ^1^ Operational Quality, Air Force Medical Agency, Falls Church, Virginia, USA.; ^2^ College of Nursing, The Ohio State University, Columbus, Ohio, USA.; ^3^ National Institutes of Health Library, Office of Research Services, Office of the Director, National Institutes of Health, Bethesda, Maryland, USA.; ^4^ Christine E. Lynn College of Nursing, Florida Atlantic University, Boca Raton, Florida, USA.; ^5^ National Heart, Lung, and Blood Institute, National Institutes of Health, Bethesda, Maryland, USA.

**Keywords:** sex and gender research, sickle cell trait, scoping review

## Abstract

Although there is an emerging body of evidence that addresses the adverse health outcomes of individuals with sickle cell trait (SCT), it is not clear if the findings are generalizable from a sex and gender perspective. The purpose of this scoping review was to complete an assessment of main concepts, identify knowledge gaps, and determine the potential value of conducting an eventual systematic review. The research question guiding this scoping review is: In SCT individuals with adverse health outcomes, what is known about sex and gender differences? We conducted a scoping review of research on SCT from 2016 to 2022 across five databases, with 36 research studies included in the review. The majority of the included studies were cohort studies (67%) in the United States (61%) focusing on a variety of health outcomes. One-third reported health outcomes by both SCT status and sex, and one-third controlled for sex; no studies addressed gender. Further research is needed on the role of sex and gender for individuals with SCT.

## Introduction

Sickle cell anemia (SCA) is the most common hematological disorder in the United States. It affects approximately 100,000 Americans, occurring in 1 in 365 African American births and 1 in 16,300 Hispanic American births.^1^ Individuals with SCA experience recurrent vasoocclusive events that lead to acute and chronic pain, and tissue ischemia, resulting in frequent visits to the emergency room or hospitalization.^2^ Multisystemic organ damage due to recurrent vasoocclusive crises causes morbidity and over the years results in a reduced life expectancy for males (49.3 vs. 73.4 years) and females (55 vs. 79.3 years) compared to the general US average, respectively.^3^

Sickle cell trait (SCT) is the carrier state for SCA and occurs at much higher rates, in approximately 1 in 13 African Americans. Although individuals with SCT are typically asymptomatic with a life expectancy aligned with an ethnically matched unaffected population SCT,^4^ the carrier state is “not completely harmless.”^5^ The American Society of Hematology acknowledges these concerns in their research priorities for SCT citing unanswered questions related to the risk for sudden death with vigorous exercise, the risk of strokes, blood clots, and heart or kidney disease, and the interaction between the inherited gene mutation and other conditions.^6^

Although there is an emerging body of evidence that addresses the adverse health outcomes of individuals with SCT, it is not clear if the findings are generalizable from a sex and gender perspective. Recent evidence suggests that both sex and gender influence different aspects of health, and disease diagnosis, treatment, and recovery. However, investigators do not always report sex and gender with the study findings. In 2016, the National Institutes of Health (NIH) implemented its policy titled Consideration of Sex as a Biological Variable (SABV).^7^ The policy highlights the need to consider sex in the research design, analyses, and report of findings, including vertebrate animals and humans, except when a strong justification exists for single-sex investigations. *Sex* is defined as genetic state, typically either XX female or XY male, and is most often reported based on one’s anatomy at birth; *gender* is a multidimensional construct based on gender identity and expression as well as individual characteristics and behaviors associated with certain sex trait and includes more than the binary concept of woman and man.^8^ Research informed by both sex and gender in design and analysis enhances the rigor and reproducibility of study findings.^9^

Because limited research has been conducted on SCT in general, it can be presumed that SABV has not been routinely considered. To further explore sex- and gender-specific research recommendations for SCT, a scoping review is appropriate. The purpose of this scoping review is to complete an assessment of main concepts, identify knowledge gaps, and determine the potential value of conducting an eventual systematic review.^10^ The research question guiding this scoping review is: In SCT individuals with adverse health outcomes, what is known about sex and gender differences?

## Methods

### Protocol and registration

A protocol was written *a priori* and published on Open Science Framework.^11^ The Preferred Reporting Items for Systematic Reviews and Meta-Analyses extension for Scoping Reviews (PRISMA-ScR) Checklist was used for reporting the completed scoping review.^10^

### Eligibility criteria

Included articles were primary research studies, published protocols, systematic reviews, and case reports written in English that addressed SCT or sickle cell carrier status, with at least one adverse health outcome variable. Adverse health outcomes were defined as disease processes other than hemoglobinopathies that may occur in the presence of SCT and were categorized as cardiovascular, kidney, infectious, and endocrine outcomes. Although specific adverse health outcomes were previously identified, this scoping review aims to explore sex and gender differences and therefore was not limited to already known disease processes. For full-text screening, sex or gender was required to be reported in the article’s results. Articles were excluded if any of the following applied: SCA only; single-sex or single-gender outcomes (*e.g.,* pregnancy, priapism); nonhuman subjects research; epidemiological studies of prevalence of SCT in populations; knowledge or awareness of SCT was the only outcome of interest; ethical or policy implications such as premarital screening; case studies in which SCT was an incidental finding; and articles that were gray literature or editorials.

### Information sources and search

A biomedical librarian (A.A.L.) searched five databases: CINAHL Plus (EBSCOhost), Embase (Elsevier), PubMed (US National Library of Medicine), Scopus (Elsevier), and Web of Science: Core Collection (Clarivate Analytics) using a combination of keywords and controlled vocabulary terms (*e.g.*, CINAHL Subject Headings, EMTREE, MeSH). The searches were completed in January 2023. The searches were limited by publication date from January 1, 2016, to December 31, 2022, and language (English); to exclude animal studies and article types specified in the eligibility criteria, two separate search strategies were used. The year 2016 was selected as the beginning of the search window as that was the first year when the NIH SABV instruction was implemented. EndNote 20 (Clarivate Analytics) was used for the collection and identification of duplicate records from the literature searches.

### Selection of sources of evidence

Screening and data extraction were completed using Covidence (Veritas Health Innovation). Titles and abstracts of all records were screened independently by two reviewers (A.K.P. and L.S.) and conflicts were resolved by group consensus discussion. Full-text screening was conducted independently by two reviewers (A.K.P. and L.S.) and conflicts were resolved by discussion with senior review team members (M.B. and C.W.).

### Data collection and data items

Data collection was pilot-tested with two reviewers and with five records in Covidence to ensure consistency in data extraction. Data were collected by two reviewers (A.K.P., L.S.) independently from each article included after full-text screening. The collected data were compared by two senior review team members (M.B., C.W.) for quality control, and discrepancies were resolved after discussion with the data collectors. The following data items were collected: purpose and country of study, methods, SCT rates by sex and gender, adverse health outcome rates by sex and gender, and any combined effects.

### Synthesis

A narrative summary of results with descriptive statistics and a table of key characteristics for included articles was planned. Key characteristics listed in the table were revised to focus on sex and gender.

## Results

**FIG. 1.**
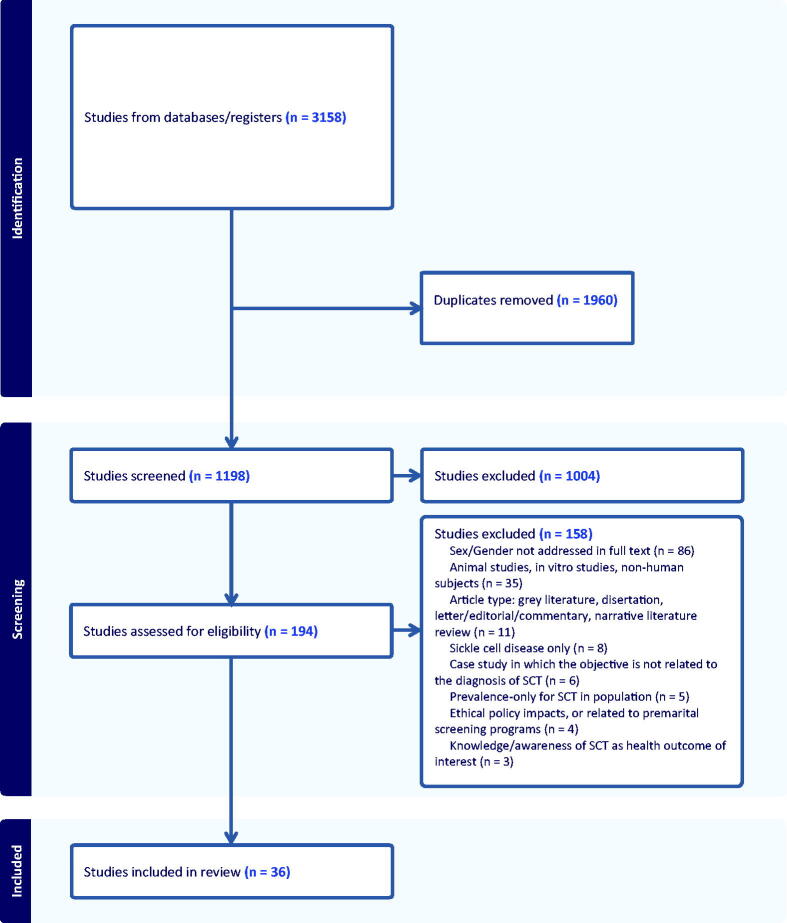
PRISMA diagram of included articles. PRISMA, Preferred Reporting Items for Systematic Reviews and Meta-Analyses.

The literature search across five databases yielded 3158 records, of which 1960 duplicates were removed, resulting in 1198 for title and abstract screening. Of those, 194 were included and proceeded to full-text screening. After completing full-text screening, a total of 36 articles were included in this scoping review (see Fig. 1).

The majority were cohort studies (*n* = 25), others were cross-sectional studies (*n* = 9), and one each of meta-analysis, case–control, and case series. There were no intervention-based studies. Twenty-four studies included only Black, African, or African American subjects (*n* = 24). The majority of studies (*n* = 22) were conducted in the United States; others were Nigeria (*n* = 4), India, or United Kingdom (*n* = 3), or other countries (*n* = 4; Uganda, Senegal, Brazil, multinational).

The methods reported to document SCT status included study-specific genotyping (*n* = 11), hemoglobin electrophoresis (*n* = 11), EMR review (*n* = 9), or chromatography (*n* = 5). The methods to document sex were not reported in the majority of studies (*n* = 21). When sex was reported, the data were collected from the electronic medical record (*n* = 9) or self-report (*n* = 6). The rate of SCT by sex was reported in 28 (78%) studies. Outcome rate was reported by SCT status in 28 (78%) studies, and by sex in 12 (33%) studies. Thirty percent (*n* = 11) of studies reported outcomes by both SCT status and sex. An additional 36% (*n* = 13) controlled for sex in the final model suggesting concern for the influence of sex but providing no specific finding to interpret. No study addressed gender implications for health outcomes.

Adverse outcomes were categorized as cardiovascular disease (*n* = 11), infectious disease (*n* = 11), kidney disease (*n* = 8), exertional heat injury (*n* = 4), endocrine disorders (*n* = 3), cognitive function (*n* = 2), HEENT (*n* = 2), and mortality (*n* = 4) (Table 1). Some articles reported on multiple outcomes (*n* = 4). For cardiovascular disease, SCT was listed as not significant for increased risk of ischemic stroke, coronary artery disease, myocardial infarction, or hypertension.^17,18,20,28,31,34,35,37,39,43,45^ No studies reported cardiovascular disease rates by sex. Of the four studies that reported sex and SCT status, there was no significant relationship between cardiovascular disease, SCT, and sex. Infectious diseases included malaria, HIV, and COVID.^14,15,19,22,23,25,26,35,36,40,44^ SCT was associated with lower rates of complications from malaria, but no sex differences were noted. HIV viral load was either not significantly increased, or was decreased with SCT status; one study showed females with SCT specifically had a lower viral load. COVID outcomes were mixed with half of the studies (*n* = 2) showing that SCT individuals had higher hospitalization rates, and the other half with SCT was not significant for hospitalization or mortality. For kidney disease, SCT status was associated with increased risk for renal dysfunction and acute kidney injury, with greater risk in males.^13,16,21,29,31,33,35,39^ Females with SCT had higher rates of exertional heat injury compared to males with SCT, but significance was not reported.^38,41^ Additionally, individuals with SCT were at higher risk for exertional rhabdomyolysis, and the majority were males.^24,47^ Although individuals with SCT may be at increased risk for endocrine disorders such as type 2 diabetes mellitus, sex differences have not been reported in our review.^12,39,43^ SCT was not associated with cognitive decline or impairment when controlling for sex, although sex differences were not reported.^30,32^ There were no significant trends in sex differences with SCT in regards to ocular hyphema or periodontal disease.^27,46^ COVID mortality was included in the category of overall mortality and is discussed above, and SCT when controlled for sex was not associated with increased mortality in a soldier population.^19,22,34,42^

**Table 1.** Articles Included in Scoping Review

First author	% M/F with SCT	Primary outcome	Outcome rate by SCT	Outcome rate by sex	Outcome by SCT and sex
2022					
Hulsizer^12^	M 35.9% F 64.1%	Type 2 diabetes mellitus (T2DM)^e^	SCT+ significant increase risk for T2DM, renal diseases, and vascular diseases OR: 1.38 (CI = 1.12–1.68)	NR	C-NI
Hung^13^	M 45.7% F 54.3%	End-stage kidney disease^c^	NR	NR	NR
Okpala^14^	NR	HIV viral load^b^	SCT+ was not significant for increased HIV viral load (*p* = 0.32).	NR	SCT+ females had lower HIV viral load (*p* = 0.018).
Patel^15^	M 42.6% F 57.4%	Tuberculosis^b^	NR	Tuberculosis M 48.4% F 51.6%	NR
2021					
Behera^16^	NR	Renal dysfunction^c^	SCT+ had clinical and laboratory markers of renal dysfunction that indicated significantly less impairment compared to SCA.	NR	NR
Hyacinth^17^	Rates vary across cohorts	Coronary heart disease and myocardial infarction^a^	SCT+ was not significant for CHD or MI. CHD HR: 1.16 (CI = 0.92–1.47) MI HR: 1.03 (CI = 0.81–1.32)	NR	C-NI
Jefferson^18^	NR	Splenic infarction^a^	NR	NR	Study population was all SCT+, 88% males vs. 12% females experiencing splenic infarction. No further analysis was done.
Merz^19^	M 55% F 45%	COVID mortality^b,h^	SCT+ was associated with higher hospitalization rates (12%) and mortality (15%)	NR	NR
Nelson^20^	Higher SCT carrier rate in females (7.8%) than in males (7.2%)	Deep vein thrombosis and pulmonary embolism^a^	NR	NR	aHR for both men and women with SCT+ was not significant for either DVT or PE. Men SCT+ DVT aHR: 0.9 (CI = 0.4–2.0) Women SCT+ DVT aHR: 1.6 (CI = 0.7–3.2) Men SCT+ PE aHR: 1.1 (CI = 0.5–2.4) Women SCT+ PE aHR: 1.2 (0.5–3.1)
Olaniran^21^	M 22% F 78%	Acute kidney injury^c^	SCT+ was significant with higher risk for AKI. aHR: 1.64 (CI = 1.27–2.11)	NR	C-NI
Resurreccion^22^	Higher SCT carrier rate in females (7.8%) than males (6.2%)	COVID rates and mortality^b,h^	SCT+ was not significant for COVID rates or mortality. COVID rates OR: 1.12 (CI = 0.66–1.84) COVID mortality OR: 2.87 (CI = 0.69–9.95)	NR	SCT+ with female sex was not significant for COVID rates or mortality. However, all individuals who died of COVID were female. SCT+/Fs COVID rates OR: 0.32 (0.08–1.38) SCT+/Fs COVID mortality OR: 0.17 (0–1.66)
Singh^23^	M 18% F 82%	COVID severe outcomes^b^	SCT+ not significant for COVID outcomes, hospitalizations, or mortality. Rates not reported.	NR	C-NI
Webber^24^	NR	Rhabdomyolysis^d^	SCT+ status was present in 3.6% of rhabdomyolysis cases. Significance is not addressed.	Rhabdomyolysis cases 92.3% male	NR
Zehner^25^	M 53.3% F 46.7%	Symptomatic malaria^b^	SCT+ was significant for lower rates of symptomatic malaria. IRR: 0.57 (CI = 0.44–0.74)	Female sex was not significant for symptomatic malaria. IRR: 1.01 (CI = 0.85–1.21)	C-NI
2020					
Balanchivadze^26^	NR	COVID outcomes^b^	Of those with SCT (18/24), 11 required hospitalization.	NR	NR
Mir^27^	NR	Hyphema^g^	NR	NR	57% M SCT with hyphema, 43% F.
Zhang^28^	M 56.4% F 43.6%	Ischemic stroke^a^	SCT+ was not significant for ischemic stroke. OR: 0.92 (CI = 0.51–1.66)	M 55% F 45%	SCT+ by sex was not significant for ischemic stroke.
2019					
Akinbodewa^29^	M 20.4% F 79.6%	Renal dysfunction^c^	SCT+ was not significant for renal dysfunction or chronic kidney disease. PR: 0.99 (CI = 0.417–2.348)	NR	Male sex was a risk factor for renal dysfunction regardless of SCT status.
Cahill^30^	M 33.4% F 66.6%	Cognitive impairment ^f^	SCT+ was not significant for cognitive impairment. aOR: 1.21 (CI = 0.92–1.60) no significant relationship to SCT when controlling for sex	45.2% M 54.8% F	C-NI
Caughey^31^	M 42% F 58%	Ischemic stroke modified by CKD^a,c^	SCT+ alone was not significant for ischemic stroke, HR: 1.31 (0.95–1.80). SCT+ with CKD was significant HR: 2.18 (1.16–4.12). SCT+ was associated with 20% higher hazard of stroke with composite outcomes.	NR	C-NI
Chen^32^	M 37.5% F 62.5%	Cognitive function^f^	SCT+ was not significant for cognitive decline.	NR	C-NI
Hu^33^	NR	Acute kidney injury and Chronic kidney disease^c^	SCT+ significant for both AKI and CKD. AKI OR: 1.74 (CI = 1.17–2.59) CKD OR: 2.00 (CI = 1.39–2.88)	Male sex significant for both AKI and CKD. AKI OR: 3.23 (CI = 2.08–5.02) M CKD OR: 1.82 (CI = 1.28–2.59) M	NR
Olaniran^34^	M 33% F 67%	Coronary artery disease, Stroke, Mortality^a,h^	SCT+ was not significant for CAD, stroke, or mortality. CAD (aHR: 1.16; 95% CI = 0.79–1.68) Stroke aHR: 0.74 (CI = 0.46–1.18) Mortality aHR: 1.52 (CI = 0.87–2.67)	NR	SCT+ interaction with sex was not significant for CAD, stroke, or mortality. CAD female SCT+ (aHR: 0.70, CI = 0.48–1.02) Stroke female SCT+ (aHR: 0.69, CI = 0.44–1.07) Mortality female SCT+ (aHR: 0.67, CI = 0.37–1.20)
Reeves^35^	M 51.2% F 48.8%	Multiple health outcomes among children^a,b,c^	SCT+ was not significant for fever, invasive pneumococcal disease, renal complications, or stroke. SCT+ was significantly less acute otitis media, acute respiratory infection, and pneumonia compared to SCT−. AOM, OR: 0.88 (CI = 0.84–0.91) ARI, OR: 0.94 (CI = 0.92–0.97) Pneumonia, OR: 0.93 (CI = 0.87–0.99)	NR	NR
2018					
David^36^	M 46.2% F 53.8%	HIV clinical outcomes and viral load^b^	Lower baseline viral load SCT+ compared to SCT− (*p* = 0.048)	NR	NR
Hyacinth^37^	Rates vary across studies in the meta-analysis.	Ischemic stroke^a^	SCT+ not significant for ischemic stroke in meta-analysis; OR: 0.80 (CI = 0.47–1.35)	NR	C-NI
Nelson^38^	M 71.9% F 28.1%	Mild heat injury and heat stroke^d^	SCT+ was not significant for mild heat injury or heat stroke. Mild heat injury HR: 1.15, CI = 0.84–1.56 SCT+ Heat stroke HR: 1.11, CI = 0.44–2.79 SCT+	Female sex was significant for mild heat injury. HR 1.76 (CI = 1.48–2.10)	NR
Niebuhr^39^	M 66.8% F 33.2%	Chronic medical conditions ^a,c,e^	SCT+ was significant for incident risk of having at least one chronic medical condition. IRR: 1.71 (CI = 1.61–1.81)	NR	C-NI
Purohit^40^	M 57.6% F 42.4%	Complications of malaria^b^	SCT+ was not significant for malaria complications anemia, hepatopathy, renal failure, jaundice, or shock. SCT− was significant for fatality from malaria compared to SCT+ (chi square: 4.33, *p* = 0.037)	Severe malaria M 69.1% F 30.9%	NR
Singer^41^	M 66.8% F 33.2%	Exertional heat injury^d^	SCT+ was significant for increased risk of exertional heat injury. aHR: 1.24 (CI = 1.06–1.45)	Female sex was significant for increased risk of exertional heat injury. aHR: 1.36 (CI = 1.17–1.59)	Higher rates of exertional heat injury in females (14.79/1000) compared to males (13.89/1000); significance not reported.
Singer^42^	M 66.8% F 33.2%	Mortality from any cause^h^	SCT+ was not significant for crude mortality ratio. OR: 1.27 (CI = 0.94–1.71)	NR	C-NI
Skinner^43^	M 25% F 75%	Hypertension in type 2 DM^a,e^	SCT+ in individuals with T2DM was significant for increased rates of hypertension, retinopathy, neuropathy, and reduced renal function (*p* < 0.05)	NR	C-NI
2017					
Ademolue^44^	NR	Malaria in children^b^	NR	Of those positive for malaria: 40% M, 60% F	Of those positive for malaria: No sex differences when stratified by genotype.
Liem^45^	M 42.7% F 57.3%	Cardiovascular risk factors^a^	SCT+ was not significant for cardiovascular risk factors. HTN 1.22 (CI = 0.91–1.65); diabetes 1.48 (CI = 0.96–2.27); metabolic syndrome 1.26 (CI = 0.92–1.74)	NR	C-NI
2016					
deCarvalho^46^	M 9% F 91%	Periodontal disease^g^	SCT+ had 53.3% with periodontal disease, compared to 27.1% SCT− (*p* < 0.001)	NR	NR
Nelson^47^	M 69.8% F 30.2%	Rhabdomyolysis^d^	SCT+ was associated with higher risk for exertional rhabdomyolysis. HR: 1.54 (CI = 1.12–2.12)	Females lower risk for rhabdomyolysis (HR: 0.51, CI = 0.38–0.67)	NR

Outcome categories:

^a^ Cardiovascular disease (*n* = 12).

^b^ Infectious disease (*n* = 11).

^c^ Kidney disease (*n* = 8).

^d^ Exertional heat injury (*n* = 4).

^e^ Endocrine disorders (*n* = 3).

^f^ Cognitive function (*n* = 2).

^g^ HEENT (*n* = 2).

^h^ Mortality (*n* = 3).

aHR, adjusted hazard ratio; AKI, acute kidney injury; CAD, coronary artery disease; CHD, coronary heart disease; CKD, chronic kidney disease; CI, confidence interval; C-NI, Controlled for Sex-Not Interpretable; DVT, deep vein thromboembolism; F, females; HEENT, head eyes ears nose throat; IRR, incidence rate ratio; M, males; HR, hazard ratio; NR, not reported; OR, odds ratio; SCT+, sickle cell trait positive (HbAS genotype).

## Discussion

This scoping review addresses the unanswered question: what is known about sex and gender differences among SCT individuals with adverse health outcomes.

One-third of studies reported health outcomes by both SCT status and sex, however, due to the variety of different outcomes reported, recommendations for any systematic reviews to explore sex differences in adverse outcomes are not feasible at this time. It should be noted that one of the studies was a meta-analysis on ischemic stroke,^37^ which found that SCT was not significant for increased risk of ischemic stroke, and controlled for sex without interpretable results. Furthermore, several studies published nonsignificant findings for SCT individuals compared to non-SCT individuals regarding health outcomes of ischemic stroke, mortality, and COVID outcomes.^14,17,19,20,22–24,28–32,34,35,37,38,42,45^ These results are important for the population, but do not contribute to the possibility of future systematic reviews. Additionally, there was a lack of intervention research, where treatment for an adverse event was reported and addressed those with SCT, and an over-representation of cohort studies which controlled for sex but did not contribute any new knowledge about the role sex or gender can play in health outcomes. Of note, the two studies that did demonstrate higher COVID hospitalization did not control for sex,^19,26^ while the two without significant findings addressed SABV specifically and that female sex with SCT was not significant for COVID.^22,23^

For studies that were excluded from this review, 86 articles were excluded because sex was not addressed in the full text to any extent. Although the purpose of this scoping review is to address the extent to which sex and gender are addressed in research about SCT, we feel it is important to highlight that a greater number of articles did not address SABV at all. The policy to consider SABV does not require sample sizes to be increased or for studies to be powered to detect sex differences, but that SABV is *considered*.^48^ There is a clear gap that needs to be addressed when SCT research is designed and conducted. Furthermore, in the 35 preclinical studies, sex of the cells or animals was not addressed. SABV should be considered in the design, data analysis, and reporting of research with vertebrate animals and humans, except when a strong justification exists for single-sex investigations.^9^

Many studies used sex and gender terms interchangeably, most frequently referring to “gender” when “sex” was the accurate term. Another scoping review found that the terms sex and gender were applied correctly in 42.5% of over 2000 articles from 1946 to 2016, where the majority considered sex as a confounding variable but did not perform additional analysis.^49^ A systematic review of reporting guidelines listed criteria for correct use of sex and gender terms including nonbinary terminology, appropriate categories, and noninterchangeability of the terms.^50^ In their review of 407 reporting guidelines, only 1 met their criteria with correct use of terminology.

No studies addressed gender differences directly. Gender differences in health outcomes for individuals with SCT may involve topics such as access to health care for diagnosis and treatment, sports involvement for concerns such as exertional heat injury, or other factors that closely align with the social determinants of health. Although we may be a long way from including future research to examine the social impacts of gender among SCT individuals, an acknowledgment of potential complications is warranted. Gender differences studies are less common than sex differences studies in medical literature.^49^

In this review, 16 of the 29 articles which reported SCT rates by sex (55%) noted more females in the study sample, or higher rates of SCT in females, depending on the context. Although SCT is not a sex-linked genetic disorder and therefore should not have any sex differences in population rates, this is worth noting as a discussion point on the intersection of sex and gender. One possible anecdotal argument is that women are more likely than men to be tested for SCT, or suggestions that women may be greater health care utilizers than men in the United States, which represented greater than 60% of the studies included in this review. Expanding education and training on SABV and requirements for sex differences research is needed on an international level.^51^

This scoping review contributes to sex differences research in the SCT population, although there are limitations. The date range for included articles was narrowed to 2016 to focus on the NIH policy, and there remains a possibility that the entire range of literature could have more breadth on SABV in the SCT population. However, prior to the NIH policy, there was limited guidance on sex and gender reporting in research. Strengths of this study included the interdisciplinary team, *a priori* protocol registration, and examination of SABV and gender as a guide for future research on SCT.

Curricula regarding sex differences should be available to all aspects of research including funding, ethical approval, and dissemination with journal guidelines and conference proceedings. As research on both SABV and gender continues to emerge, there may be potential to determine if women and men with SCT and a health condition would have different outcomes. As research in this area continues to develop, a systematic review of sex and gender differences is a goal for future research.

## Abbreviations Used

EMR: electronic medical record
NIH: National Institutes of Health
SABV: sex as a biological variable
SCA: sickle cell anemia
SCT: sickle cell trait
